# Hospitalization cost of conventional psychiatric care compared to broad-spectrum micronutrient treatment: literature review and case study of adult psychosis

**DOI:** 10.1186/s13033-017-0122-x

**Published:** 2017-01-31

**Authors:** Bonnie J. Kaplan, Wanrudee Isaranuwatchai, Jeffrey S. Hoch

**Affiliations:** 1 Cumming School of Medicine, University of Calgary, Alberta, Canada; 2 Centre for Excellence in Economic Analysis Research, St. Michael’s Hospital and Institute of Health Policy, Management and Evaluation, University of Toronto, Ontario, Canada; 3 Department of Public Health Sciences, University of California, Davis, USA

**Keywords:** Mental healthcare utilization costs, Nutrient treatment, Psychosis

## Abstract

**Background:**

Healthcare costs are skyrocketing, with mental health treatment amongst the most expensive, especially when hospitalization is involved. According to the Mental Health Commission of Canada, one in five Canadians is living with a mental disorder in any given year, at an annual cost of $50 billion. In light of this societal burden, alternative approaches are being evaluated, such as brief psychotherapy by phone, peer support, and, as part of the emerging field of nutritional mental health, treatment with micronutrients (minerals and vitamins). Effectiveness of micronutrients has been demonstrated for many types of psychiatric symptoms, in about 45 studies of formulas that are either multinutrient (e.g., several B vitamins) or broad-spectrum (usually over 20 minerals and vitamins). Although this literature demonstrates therapeutic benefits, the potential economic impact of micronutrient treatment has been evaluated in only one case study of childhood psychosis.

**Methods:**

The current case study was initiated to evaluate mental health-related hospitalization costs from 1997 to 2003 for a female adult diagnosed with various mood and psychotic symptoms. She was treated for the first 5 years with conventional methods and then subsequently with a broad-spectrum micronutrient formula.

**Results:**

The patient’s annual mental health hospitalization costs during conventional treatment averaged $59,864 across 5 years (1997–2001), with a peak annual cost of about $140,000. Since transitioning to broad-spectrum micronutrients, she has incurred no provincial hospitalization costs for mental health care, though her self-funded costs are currently $720/year for the micronutrients.

**Conclusion:**

Further exploration of the treatment of mental health problems with broad-spectrum micronutrient formulas has the potential to make two significant contributions: improved mental health, and decreased costs for governments.

## Introduction

Most societies are concerned about the ever-increasing costs of healthcare, especially for mental disorders. In Canada, where it is estimated that one in five people experience mental illness in any given year, the annual direct cost of mental health treatment currently exceeds $50 billion and is expected to rise to over $100 billion in the 2020s [1]. Alternative approaches are being evaluated with increasing frequency, including various types of psychosocial and peer support which may prevent subsequent expensive hospitalization or long-term pharmaceutical costs [2]. Another type of mental health treatment whose effectiveness is increasingly reported in the medical literature involves the use of minerals and vitamins (together referred to as micronutrients).

This paper briefly reviews the research on micronutrient treatment of mental health disorders, including the biologic rationale for this approach, and then summarizes a previously published pediatric case study of psychosis which provided a comparison of the health cost of conventional psychiatric care versus micronutrient treatment. Subsequently, a new case study of psychosis is presented, evaluating hospitalization costs of an adult treated first with conventional psychiatric care for five years (1997–2001), and then with micronutrients (2002–present).

## Background

### Micronutrient treatment of mental health disorders

In the past 15 years, there have been over 25 reports of mental health benefits from very broad-spectrum formulas consisting of more than 20 minerals and vitamins [3]. If one adds these results to the number of studies of multinutrient formulas consisting of just a few minerals or vitamins (e.g., the evaluation of B complex formulas), there are approximately 45 empirical reports, at least 25 of which were randomized controlled trials (RCTs), and all of which are recent [3]. The following is a review of the use of broad-spectrum mineral–vitamin combinations for treating several individual mental symptoms and disorders.

Aggressive behaviors and conduct violations have been studied in preadolescent and adolescent students in working-class public schools (two RCTs) and in incarcerated adolescents and young adults (three RCTs). All five RCTs of broad-spectrum micronutrient treatments (involving 23–25 micronutrients) have shown marked effects on major disciplinary violations and on aggressive behavior, with 26–47% reductions in the frequency of conduct violations [4–8]. In three of these studies, the formulations included omega fatty acids, but two did not and the results were similar. Although these five positive RCTs (with no negative RCTs) are probably sufficient to justify implementation in correctional settings, changing policies in such institutions can take many years.

Attention deficit hyperactivity disorder (ADHD) appeared to be effectively treated with broad-spectrum micronutrients in one RCT in adults and nine open-label studies in youth and adults. The RCT with adults included 80 individuals [9]: micronutrients produced greater changes than placebo in both hyperactivity/impulsivity (intent to treat effect sizes (ES) of 0.46–0.67) and inattention (ES 0.33–0.62) on both patient and family ratings; clinician ratings on individual items were mixed but revealed greater improvement in overall psychiatric functioning on micronutrients than placebo (ES 0.53–0.57). Two open-label within-subject crossover studies (using ABAB designs) of 8–15 year olds with ADHD showed significant on–off symptom control on measures of ADHD, conduct, and mood [10, 11].

Mood disorders have shown improvement in 16 open-label reports in adults and youth with bipolar disorder and major depressive disorder (MDD) [reviewed in 9, 12]. After 4–8 weeks of treatment with broad-spectrum micronutrient formulas, approximately 50–80% of clients with bipolar disorder displayed better scores on measures of mania and/or depression. In addition, most patients taking conventional drugs were able to completely discontinue them (with follow-up for at least 6 months). No RCT has yet been reported that selected participants specifically for MDD, but post hoc analysis of one trial with adults with ADHD [9] examined a subsample of participants who met criteria at baseline for major depression. Among the 21 adults with moderate or severe depression at baseline (Montgomery–Asberg depression rating scale score ≥20), the observed antidepressant effect of broad-spectrum micronutrients was substantial (ES 0.64, p < 0.04). Although still preliminary, it is important to note that these post hoc findings suggest antidepressant benefits comparable to the effects of conventional antidepressants. An open-label study of 10 preadolescents with bipolar disorder showed a 45% decrease in mania scores and a 37% decrease in depression scores on intent-to-treat analysis [13]. These findings are clearly promising, and dedicated RCTs on subjects recruited for mood disorders are needed.

Mood in nonclinical samples has been examined in many studies, with no consistent effects at recommended daily allowance (RDA) dosing levels. However, a meta-analysis found that formulations with higher doses of B vitamins had more effect on mood ratings—especially when the vitamin B doses were 5–10 times above RDA levels [14].

Anxiety and resilience to stress. At least 10 RCTs have demonstrated that broad-spectrum micronutrients reduced stress and anxiety in nonclinical sample; i.e., “normal” people in a variety of situations, such as the aftermath of natural disasters. Two RCTs conducted after earthquakes [15] and floods [16] showed reduced acute stress and anxiety scores; in one [15], the prevalence of probable post-traumatic stress disorder (PTSD) decreased from 65 to 19% after one month of treatment, while the control group remained unchanged. These two RCTs suggest that broad-spectrum micronutrients could be an inexpensive public health intervention for normal populations following natural disasters.

In summary, there is evidence for the beneficial effects of broad-spectrum micronutrients on diverse mental health targets, although much more research is needed. The strongest evidence includes RCTs on violent and nonviolent conduct problems, as well as on ADHD. In addition, there are strongly suggestive data on mood disorders, as well as some additional reports on anxiety disorders, obsessive–compulsive disorder, autism spectrum disorder, and substance abuse disorders [3].

In contrast, single micronutrients show limited promise, and only as augmentation agents (not as monotherapies) [17]. In other words, broad-spectrum micronutrients appear to have greater potential to modify central nervous system function in more pervasive and potent ways.

### Biologic rationale of micronutrient treatment

Micronutrients are essential throughout the central nervous system to ensure optimal function [18]. One way in which they fulfill this role is to serve as cofactors for the enzymatic activity required in every metabolic step involving synthesis of neurotransmitters. They also feed our mitochondria, present in every cell in our brain and body, thereby providing an essential function in combatting excessive oxidative stress and inflammation [18]. Micronutrients are also required for regulatory proteins such as transcription factors that regulate gene expression and epigenetic effects. As just one example of how pervasive their functions are, the dietary mineral zinc has defined roles in over 100 types of enzymes that are required in the mammalian brain for optimal metabolism.

There have been numerous reports demonstrating micronutrient deficiencies in the modern diet, in part due to the over-reliance on highly processed foods. And there is a strong consensus from nutritional epidemiology studies that people with higher intakes of highly processed food have more symptoms of depression and anxiety; in some longitudinal analyses, the dietary pattern of highly processed food intake has been shown to precede onset of the psychiatric disorders, indicating that the way we eat may be a risk factor for mental health disorders [19].

### Pediatric case study comparing costs of conventional vs micronutrient treatment

Although two published case studies of psychosis have demonstrated successful remission of symptoms with broad-spectrum micronutrient formulas [20, 21], only one of those determined the potential economic impact of treating mental health problems in this manner [21]. That case was a child aged 10 diagnosed with increasingly severe anxiety, then obsessive–compulsive disorder (OCD), psychosis with visual and auditory hallucinations, and delusions. As an inpatient for six months in a specialized pediatric mental health unit at a tertiary care hospital, he was trialed on many psychiatric medications, resulting in no improvement: at entry, his Children’s Global Assessment Scale score was 35 out of 100 (indicative of very low functioning), and at discharge, it was still 35 out of 100. At the end of 6 months, the child was sent home and referred to outpatient psychiatric care, where his parents requested that he be treated with one of the broad-spectrum micronutrient formulas^1^ that had been studied. Daily ratings were collected during the following year from the boy’s parents (who evaluated symptoms of OCD and psychosis) and from the child himself (who evaluated his own auditory and visual hallucinations). A cross-tapering protocol was followed (increasing nutrients while decreasing medications), and by one month, the child was medication-free. By 6 months, most of his symptoms were gone and he was leading a fairly normal life. A year later, he was discharged from the outpatient clinic with a significantly improved score of 70 on the Children’s Global Assessment Scale; all diagnoses had remitted. This child has been followed now for seven years, and he remains symptom-free and he is compliant with taking his nutrient capsules. In May 2015, he graduated from high school and he now holds a part-time job.

A health economist retrieved the provincial healthcare costs for the child’s six months of inpatient care, as well as for the six months of outpatient care immediately following his discharge while he was taking micronutrients. The cost of micronutrients and some outpatient visits in the second 6 months was <2% of the prior 6 months of inpatient treatment. The important difference, of course, was the remission of psychiatric symptoms while taking micronutrients (a result not seen in the medication trials). However, a second notable difference was that the much larger cost of inpatient care was incurred by the provincial health care system, while his ongoing, successful treatment with micronutrients is managed by the family at a cost of about $150/month.

This child’s positive response while taking micronutrients was consistent with the published clinical trials [3, 12], and the cost analysis added to our understanding of the potential societal benefit of exploring this treatment option. We present here a new case report that compares the costs incurred by an adult treated initially with conventional medications and later with micronutrients.

## Current case report

### History

“Marie” was first treated for a psychiatric illness at the age of 33 in February 1997 in the province of Ontario. She had been adopted at 3 months of age and was later told that substance use issues, mental health challenges, and suicide were reported in her biological relatives. Marie describes her childhood as having been associated with hyperactivity, but as an adult, she was a very high functioning and hardworking business executive. She left her job in December 1996 as her mental health deteriorated. The psychiatric reports indicate she had not previously missed work for any psychiatric reasons. By February 1997, she was having periods of psychosis for which she was hospitalized.

Marie’s diagnoses from 1997 to December 2001 (when she began micronutrients) included bipolar disorder (with psychotic symptoms), schizoaffective disorder, depression, and post-traumatic stress disorder. This variety of diagnoses is not unusual for a serious psychiatric problem that is treated in several locations over a period of several years. The psychiatric reports indicate she spent most of those years in and out of hospital; Marie herself estimates she was an inpatient about 70% of the time.

## Methods

Marie contacted one of the study authors (BJK) to request that this cost analysis be carried out, as she has spoken publicly about her struggles with mental challenges, and her perception of wellness attained by using micronutrient treatment. She provided her health care numbers for each of the provinces in which she resided from 1997 to the present, and she signed a consent form approving the work and its publication. Consultation was sought regarding the appropriateness of a research ethics review, and the University of Calgary’s Conjoint Health Research Ethics Board advised that it would not be necessary or appropriate, consistent with the ruling of the federal Secretariat on Research Ethics that “an interesting clinical case would not fall within the definition of research in the second edition of the Tri-Council Policy Statement: Ethical Conduct for Research Involving Humans (TCPS 2), to the extent that these reports are simply anecdotal accounts of individual cases.” Ultimately, this study was reviewed and approved by St. Michael’s Hospital’s Research Ethics Board.

The purpose of this analysis was to examine hospitalization costs for mental health care from 1997 to 2003 for a woman with serious mental illness. From 1997 to 2001 she received only conventional treatment. In December 2001, she transitioned to a broad-spectrum micronutrient formula and off of all conventional psychiatric medications.

We conducted a secondary data analysis focused on this case study. Data on hospital admission and cost were obtained from the discharge abstract database (DAD) at the Canadian Institute for Health Information (CIHI). With the data, we examined the trend of hospitalization cost with respect to the received treatment over time in Canada from the perspective of public health care payer (i.e., Ministry of Health). Costs are reported in 2015 Canadian dollars.

## Results

**Fig. 1:**
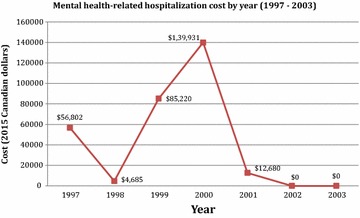
Mental health-related hospitalization cost by year (1997–2003). Conventional care was provided 1997–2001. Micronutrient treatment began December 1, 2001

From 1997 to 2003 (the study period), our case study’s residence included Ontario (1997–2003), British Columbia (1998), and Alberta (2002–2003). Based on the data availability, we focused our analysis mainly on hospitalization cost and excluded outpatient costs of psychiatric appointments, medications, etc. The findings showed that the case study’s hospitalization cost from 1997 to 2001 ranged from $4685 to $139,931 per year, with the average over 5 years being approximately $59,864. Figure 1 shows the total hospitalization costs from 1997 to 2003.

Excluded from the Figures and analyses were three hospitalizations not related to mental health: in 2001 Marie underwent a urinary tract/bladder procedure, and in January 2003 she was hospitalized twice for pregnancy-related treatments (the second of which was the birth of her son).

On December 1, 2001, Marie began taking a micronutrient formula and reports that she has not required hospitalization for psychiatric care since then. A psychiatric report dated April 1, 2005 (prepared as part of a civil claim for unjust dismissal) described her use of the micronutrient formula this way: “it is probably true that it is the most effective single treatment which [Marie] has tried.” At the present time, Marie reports that she takes an average of five capsules/day, and that she does “…notice a difference in my attitude (much more positive) and efficiency to deal with stress, when on nutrients. I am just a happier person.”

As many others do, Marie now adjusts her dosage for her stress level, so her cost for the micronutrients currently (2016) is about $60/month ($720/year). She is currently employed and is actively involved in volunteer work on matters related to national mental health initiatives.

Another cost evaluation that may be informative is to compare Marie’s annual current cost of $720 for micronutrients to a typical cost of outpatient care for mental health problems. In the study by Simpson and colleagues cited above [2], patients being discharged from hospital were randomized to either three months of an outpatient peer-support treatment program or to treatment as usual, consisting of care from mental health services. Since they did not find a group difference, we pooled their costing data for both groups over three months to compare to Marie’s annual cost of about $720. Taking the costs they provided for three months, extrapolating to twelve months, and converting from British pounds in order to compare to the current case report, we found that annual costs ranged from $17,152 to $27,796 (depending on whether missing data were excluded or imputation procedures were employed). Based on the average of those two ($22,474), we can conclude that the cost of micronutrient treatment is approximately 3% of an individualized peer support outpatient program; this estimate is remarkably similar to the 2% calculated in the previously published case report [21].

## Discussion

Since beginning the micronutrient formula in December 2001, Marie’s healthcare costs have decreased: she has not required hospitalization for psychiatric problems, she receives no outpatient psychiatric treatment, and she takes no psychiatric medications. These findings are consistent with the previously-published cases of two different children with severe psychotic disorders [20, 21]. Marie continues to pay for her micronutrients herself as there is no public coverage. The cost of the micronutrients on an annual basis is <10% of the annual cost of hospitalization during the 5-year period of conventional treatment from 1997 to 2001.

The benefits of the micronutrients appear to be long-lasting. The two children previously reported [20, 21] have been followed for eight and seven years, respectively, and Marie has now been well for 15 years on the micronutrients. The fact that the reported benefits have been sustained is consistent with other published data demonstrating long-term benefit [22, 23], as well as logic: it is difficult to imagine that human metabolism would ‘habituate’ to the micronutrients which we have evolved to require and which constitute the foundation of our need to consume food on a daily basis.

Case studies have limitations, not the least of which is the fact that they fall low in the hierarchy of levels of evidence. Nevertheless, much can be learned from analyses such as the present one, and *over*-reliance on RCTs is now a recognized problem in the medical literature [24].

**Fig. 2:**
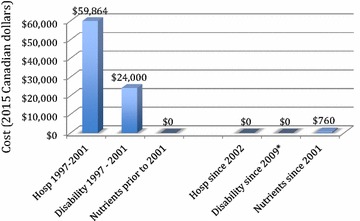
Annual costs for hospitalization, disability payments, and micronutrients. Hospitalization costs are based on data from Canadian Institute for Health Information (CIHI). Costs for provincial disability payments and self-funded micronutrients are patient-reported estimates. *Disability payments continued until 2009 as explained in the text

Another limitation in the current analysis is that there was little information available on healthcare utilization outside of inpatient hospitalization. For instance, there were four visits to emergency rooms detected in Alberta, for a total cost of less than $40. Those were not included in the findings. Our analysis focused mainly on hospitalization cost from the Ministry of Health’s perspective. There may be additional costs which the analysis missed; for example, costs associated with productivity loss and outpatient physician visits which Marie reports were often weekly for about 5 years. Finally, the disability pension cost illustrated in Fig. 2 is based on the patient’s report that she received about $2000/month during this period. Given the seriousness of her previous mental health problems, her family convinced her not to return to work for several more years, which is why the disability costs displayed in Fig. 2 extended into 2009. All in all, we suspect our estimate is a conservative estimate of the cost savings to the system in this one case.

## Conclusions

There is now a rich literature showing the effectiveness of micronutrient treatment of mental health disorders for a range of symptoms [3, 12]. Included in this literature are two prior cases of psychotic disorders [20, 21], and in one of those the use of micronutrients resulted in <2% of the cost of conventional inpatient mental health treatment. The current case report adds to the literature on this topic, showing that micronutrients are far less expensive (and apparently more effective in some cases) than conventional care.

There are at least two reasons for policy makers in health care systems to take note of these accumulating findings. First, because there is no standard insurance or government reimbursement for broad-spectrum micronutrient formulas right now, there are many people who do not take them because of the cost that they must personally absorb. This is unfortunate because the large effect sizes reported in the various group studies indicate that there is significant potential, but this potential benefit is being missed. Second, it is clearly less expensive for governmental healthcare systems to cover the costs of micronutrient therapy than to pay for conventional care (e.g., hospitalization, psychiatric appointments, and medications). This opportunity for financial savings would allow the cash-strapped healthcare system to cover other much-needed care.

## Patient’s perspective

The following statement was written by Marie, looking back on her mental health over the past 20 years:

“I have survived an overwhelming and dangerous journey of treatment, only to discover that what I actually needed was to feed my brain and recognize I was having a normal reaction to trauma and stress. It may sound strange to some people, but the truth is that I am well because I did *not* do what my doctors told me. I did not accept their diagnoses, labels, opinions or treatment, and there were many over the years. Ultimately, I did it my way and I’m alive because of it.

The important questions for me are these: (1) Why, when we know micronutrient therapy can help bring mental and emotional wellness, do we continue to ignore it in our system of health care? (2) Why, when we know the possibilities of healing with nutrition, and that it is safe and non-addictive, do we not choose this path of treatment *prior to* drugs that can be damaging and addictive?

Both questions boil down to the same thing: why, within a so-called educated society and mental health care system, do we choose to invest in the most dangerous and expensive protocol first? This is the billion dollar question.”

## Abbreviation

RCT: randomized controlled trial

## Footnotes

1 The formula used in all three case studies is EMPowerplus, marketed by Truehope Nutritional Support. Variations of this formula, all of which have been developed by two companies in Alberta, Canada, have been sold as EMPower, EMPowerPlus, EMPowerPlus Advanced, Truehope EMP, Q96, and Daily Essential Nutrients.
